# Pediatric thyroid cancer guidelines: challenges in stratifying care based on limited data

**DOI:** 10.1530/ETJ-22-0180

**Published:** 2022-10-13

**Authors:** Andrew J Bauer, Jonathan D Wasserman, Steven G Waguespack

**Affiliations:** 1 Division of Endocrinology and Diabetes, The Children’s Hospital of Philadelphia, Department of Pediatrics, The University of Pennsylvania, Philadelphia, Pennsylvania, USA; 2 Division of Endocrinology, The Hospital for Sick Children, Department of Pediatrics, University of Toronto, Toronto, Ontario, Canada; 3 Department of Endocrine Neoplasia and Hormonal Disorders and Department of Pediatrics-Patient Care, University of Texas MD Anderson Cancer Center, Houston, Texas, USA

The 2022 European Thyroid Association Guidelines for the management of pediatric thyroid nodules and differentiated thyroid carcinoma (1) were developed by a task force comprising well-respected, expert clinicians with good representation of the European community, including several previously involved in authorship of country-specific guidelines from Poland (2), the United Kingdom (3), the Netherlands (4), and the United States (5). Van Santen and the guidelines committee are to be commended for their thoughtful and stringent review of the literature in an effort to optimize the care of pediatric patients diagnosed with thyroid nodules and thyroid cancer.

The authors used the 2015 American Thyroid Association (ATA) Pediatric Thyroid Nodule and Differentiated Thyroid Cancer Guidelines as the framework and built upon these through a rigorous literature review, including the application of PICOTS methodology (6) (defined by the European Thyroid Association (ETA) as ‘population, etiologic/risk factor, comparison, outcome, type of question, and study design’) to answer questions pertinent to the care of children with thyroid neoplasms. The transparency and completeness of the literature review and the recommendation to centralize care at high-volume centers are important strengths of the guidelines. Several additional contributions in these guidelines include suggesting a minimal interval of 1 year to repeat ^131^I therapy and the recommendation that serial neck ultrasound and serum thyroglobulin measurements are sufficient surveillance for the majority of patients with differentiated thyroid carcinoma (DTC). Additional suggestions for which there are minimal existing pediatric data include the potential use of recombinant human thyroid-stimulating hormone for radioactive iodine (RAI) imaging and therapy, reducing the minimum time on a low-iodine diet to 4 days prior to ^131^I, and modifying the recommendation for lifelong surveillance from the 2015 ATA Pediatric Guidelines to ‘at least 10 years’.

Importantly, the ETA Guidelines remain predicated on a treatment paradigm that has not significantly changed in decades, including a reliance on RAI, recommending or suggesting that (1) the majority of patients undergo total thyroidectomy and that lobectomy is sufficient only for a *post hoc* incidentally found small cancer, (2) prophylactic central neck dissection should be limited to patients with ‘advanced’ thyroid cancer, (3) all children should undergo postoperative staging that includes measurement of Tg and ^131^I-post-therapy scintigraphy without a diagnostic scan, and (4) somatic molecular testing does not play a role in the evaluation of pediatric thyroid nodules or DTC management. While these guidelines advocate treatment stratification according to perceived benefit, notable exclusions from these guidelines are a tiered risk stratification at diagnosis to identify those patients at highest risk for persistent or distantly metastatic disease and the recommendation for incorporating data from postoperative staging to determine the appropriate candidates for therapeutic RAI, an approach that has been evolving in our practices over the last decade (7).

As current cochairs of the ATA Pediatric Thyroid Nodule and Differentiated Thyroid Cancer task force, we empathize with the challenges inherent in developing guidelines that incorporate practice variations reflecting geographic differences in the incidence of pediatric thyroid cancer, variable access to health care, differing cultural norms, and disparate local and regional resources across the breadth of our global pediatric thyroid community. As previously reported (8), the dispersed care and differing clinical practices is a problem for the international community. Moreover, we are acutely conscious of the fundamental benefit of providing practice recommendations based on high-quality data. While the gold standard for this is the prospective, multicenter randomized trial, the rarity of thyroid cancer in children, the widely distributed nature of care, the slow pace of disease, and a lack of adequate research funding have proven to be obstacles to conducting such studies. As a consequence, the bulk of extant data related to pediatric thyroid cancer mostly derives from single-center retrospective studies.

Highlighting this limitation is the observation that the ETA Guidelines base 52/62 (84%) suggestions or recommendations on very-low quality (grade 4) studies, including case reports and expert opinion. Similarly, in the ATA Guidelines, 76% of recommendations were based on fair-quality evidence or expert opinion, thus highlighting the reality in which practitioners function when treating rare conditions in the real world. This also raises the question as to whether or not guideline methodologies such as GRADE (Grading of Recommendations Assessment, Development and Evaluation) (9) are relevant to conditions where the disease rarity limits the ability to conduct study designs of the highest standard. Guideline task forces are thus forced to decide whether to base recommendations solely on existing studies or to deviate from published literature to innovate and to address practice needs for which no (or inadequate) literature exists. Incorporating expert opinion that draws on the experience of clinicians focused on the care of thyroid neoplasms and broader inclusion of both pediatric and adult data, when applicable, is the only currently available path to help fill in the gaps until high-quality data exist to address crucial areas of management. In the end, we all have the same goals: *primum non nocere* and to advance patient-specific stratification of care for children diagnosed with a cancer recognized to have a very low disease-specific mortality and a high rate of therapeutic complications. As pediatric thyroid centers of excellence (as advocated in the ETA Guidelines) continue to emerge, an increasing cadre of expert clinicians with experience in the breadth of pediatric thyroid disease may be leveraged to provide ‘best practice recommendations’ in areas where high-quality studies are yet to be conducted.

The ATA Pediatric Guidelines are presently undergoing a major revision. An overarching principle of this update is to provide clinicians with additional avenues to individually stratify care based on the invasiveness of disease at presentation and during continued surveillance using dynamic risk stratification (10, 11). This empowers providers to deintensify care and avoid overtreatment of children with low-risk disease while facilitating the identification and care of patients for whom more comprehensive and/or innovative therapies may be warranted. In order to better aid clinicians on the frontlines and to advance the field, even in the absence of rigorous clinical studies, priority goals in any contemporary guidelines development should be to: (1) refine the evaluation of thyroid nodules, providing specific recommendations for the use of ultrasound to identify nodules that may be monitored versus those that should undergo fine-needle aspiration, (2) refine stratification of thyroid nodules with indeterminate cytology (incorporating the option for oncogene analysis in select cases based on accumulating knowledge of pediatric PTC genomics), (3) reevaluate the surgical approach and better identify patients who would benefit from thyroid lobectomy instead of total thyroidectomy, (4) provide additional granularity to the ATA low- and intermediate pediatric strata for predicting residual/recurrent disease, (5) delineate better the patients who should not routinely be treated with ^131^I, even in the presence of lymph node disease, and (6) in the current era of tumor genomics, define patients in whom somatic molecular testing may be important in the management of their disease with RAI and the newly available selective targeted kinase inhibitors, not only in the setting of traditional RAI refractory disease but also in the neoadjuvant setting.

With the recent publication of multiple pediatric thyroid nodule and DTC guidelines, our hope is that we can better harness our collective experience to harmonize pediatric thyroid cancer care across the world. Efforts are underway to formalize our global pediatric thyroid community. The Child and Adolescent Thyroid Consortium, an expanding collaborative effort between pediatric thyroid centers throughout North America, provides one such step. Aligning registry data, formalizing our commitments to build a global community, establishing forums to share data, aligning guidelines (adjusted based on regional resources and norms), and developing clinical and research protocols will help to accelerate the field of pediatric thyroidology.

Again, we applaud the incredible effort of the members of the ETA who have worked tirelessly, despite the lack of high-quality data, to harmonize approaches to management of children with thyroid nodules and DTC across the European continent. It is through our ongoing international collaborative efforts that we can continue to move the field two steps forward without taking a step back.

## Declaration of interest

The authors declare no conflict of interest associated with the formation of this article. This commentary is a personal opinion of the co-authors and may not reflect the opinion of the ATA Pediatric Guideline revision task force.

## Funding

This work was supported by The Children's Hospital of Philadelphia Frontier Program (AJB).
